# Cracking the code of cancer immunotherapy resistance: emerging roles of pyroptosis and necroptosis

**DOI:** 10.1186/s13046-025-03569-3

**Published:** 2025-11-20

**Authors:** Kunming Lei, Jiujiu Chen, Yanrong Deng, Yige Peng, Xiang Zhai, Xianghai Ren, Jianhong Zhao, Baoxiang Chen, Congqing Jiang

**Affiliations:** 1 Department of Colorectal and Anal Surgery, Zhongnan Hospital of Wuhan University, Wuhan, China; 2 Hubei Key Laboratory of Intestinal and Colorectal Diseases, Zhongnan Hospital of Wuhan University, Wuhan, China; 3 Clinical Center of Intestinal and Colorectal Diseases of Hubei Province, Zhongnan Hospital of Wuhan University, Wuhan, China; 4 Low Rectal Cancer Diagnosis and Treatment Center, Zhongnan Hospital of Wuhan University, Wuhan, China; 5 Wuhan Clinical Research Center for Constipation and Pelvic Floor Disorders, Zhongnan Hospital of Wuhan University, Wuhan, China; 6 Rosalind and Morris Goodman Cancer Institute, McGill University, Montreal, QC H3G0B1 Canada

**Keywords:** Cancer immunotherapy resistance, Immunogenic cell death, Pyroptosis, Necroptosis, Tumor microenvironment

## Abstract

Therapeutic resistance and recurrent metastasis continue to pose major obstacles in the treatment of malignant tumors worldwide. Immunogenic cell death (ICD), characterized by its ability to both eliminate cancer cells and stimulate antitumor immune responses, has emerged as a promising strategy in the field of cancer immunotherapy. As key subtypes of ICD, pyroptosis and necroptosis contribute significantly to remodeling the tumor microenvironment (TME) and modulating immune responses through their distinct death-immunity coupling mechanisms. Characterized by plasma membrane pore formation and subsequent release of cytoplasmic contents, pyroptosis and necroptosis reprogram the immune microenvironment, thereby laying the groundwork for enhanced antitumor immune responses. Paradoxically, the chronic activation of pyroptosis and necroptosis pathways may contribute to cancer progression. Sustained inflammation within the TME promotes the release of pro-angiogenic and immunosuppressive factors, driving myeloid-derived suppressor cells (MDSCs) recruitment, extracellular matrix remodeling, and metastatic niche formation, thereby facilitating tumorigenesis and metastasis. The context-dependent dual roles of pyroptosis and necroptosis—shaped by tumor histotype, chronic inflammation, and stromal context—highlight the need for a nuanced understanding of their tumor-specific functions across cancer types. This review outlines the underlying mechanisms of pyroptosis and necroptosis, and summarizes recent advances, aiming to inform and inspire novel strategies in overcoming cancer immunotherapy resistance.

## Highlights

- This study focuses on the intricate interplay between cell death and cancer immunotherapy resistance, aiming to address the critical clinical challenge of cancer immunotherapy.
- This article systematically reviews the distinct mechanisms underlying pyroptosis and necroptosis across diverse cancer types, highlighting their influence on the tumor immune microenvironment and their context-dependent, double-edged roles in modulating antitumor immunity.
- The present study systematically summarizes emerging therapeutic strategies that induce pyroptosis or necroptosis to overcome immunotherapy resistance, including small-molecule agents, nanoplatforms, and novel molecular targets across various cancer types.

## Introduction

The global burden of malignant tumors continues to escalate, presenting persistent public health challenges that are exacerbated by population aging and environmental changes. According to the epidemiological analyses by the International Agency for Research on Cancer, global cancer incidence has increased by more than 50% over the past three decades [1]. While hereditary susceptibility, environmental carcinogen exposure, and lifestyle-related risk factors constitute primary etiological drivers, tumor therapeutic resistance and metastatic recurrence—fueled by tumor heterogeneity—persist as critical barriers in clinical oncology. Despite remarkable advancements in precision oncology, including minimally invasive surgical techniques, molecularly targeted therapies, and immune checkpoint inhibitors (ICIs), the five-year survival rate for metastatic malignancies remains below the critical threshold of 30% [2]. Although ICIs have revolutionized the antitumor immunity against malignancies, a substantial proportion of patients still exhibit primary or acquired resistance [3]. One contributing factor partly stems from defective immunogenic cell death (ICD), a critical process for generating tumor-specific T-cell responses. Suppression of this cell death pathway contributes to the formation of clinically non-inflamed “cold tumors”, which are characterized by inadequate dendritic cells (DCs) activation, T-cell exclusion, and an immunosuppressive microenvironment [4]. These findings highlight the potential of harnessing ICD induction as a promising strategy for cancer immunotherapy.

Programmed cell death (PCD), encompassing apoptosis, pyroptosis, necroptosis, and other regulated necrotic pathways, is a gene-encoded and strictly controlled process that maintains cellular homeostasis by selectively eliminating senescent, damaged, or transformed cells through intrinsic surveillance mechanisms [5]. As a distinct subtype of PCD, ICD has garnered considerable interest in cancer immunotherapy for its robust immune-activating potential. ICD links cell demise to antitumor immunity through a cascade of signaling events and the release of immunostimulatory cellular contents [6]. Among the various forms of ICD, pyroptosis and necroptosis induce cell death via plasma membrane pore formation while simultaneously triggering immune activation, establishing unique “death-immunity” linkages that can be harnessed therapeutically. Emerging evidence indicates that dysregulated pyroptosis and necroptosis are key modulators of immune evasion.

Pyroptosis, a pro-inflammatory cell death, is characterized by cell swelling, membrane perforation, and release of intracellular contents. First proposed in 2001s and mechanistically clarified following the identification of the gasdermin protein family in 2015s, pyroptosis is mediated by gasdermin pore formation [7, 8]. This process releases cytokines and damage-associated molecular patterns (DAMPs) such as IL-1β and IL-18, which activate DCs and cytotoxic T-cells (CTLs) [9, 10]. Critically, the potent inflammatory cascade and DAMPs release triggered by pyroptosis can directly counteract the immunosuppressive features of “cold” tumors, thereby offering a mechanistic strategy to circumvent resistance to immunotherapies like ICIs.

Necroptosis, first observed in the 1990s and formally identified in 2000s as a programmed form of necrosis distinct from classical apoptosis, was established as a unique mode of cell death in 2012s with the discovery of MLKL-mediated pore formation on the plasma membrane [11–13]. This pathway relies on the RIPK1–RIPK3–MLKL axis to induce membrane rupture and the subsequent release of immunostimulatory molecules such as HMGB1 and ATP, thereby enhancing antigen presentation and inflammatory responses via signaling cascades like TLR4/NF-κB [13, 14]. Similar to pyroptosis, the robust immunogenicity induced by necroptosis through the release of potent DAMPs like HMGB1 and ATP holds significant promise for converting immunologically “cold’’ tumors into “hot” ones, thereby sensitizing them to immunotherapy and overcoming existing resistance mechanisms [15, 16]. Despite the transformative impact of cancer immunotherapy on malignant tumor treatment, therapeutic resistance remains a major constraint on its therapeutic efficacy. Clinically, immunotherapy resistance is broadly categorized into two core types with distinct clinical profiles and molecular mechanisms: primary resistance and acquired resistance [17, 18]. Primary resistance manifests as an initial lack of response to immunotherapy, frequently observed in immunologically “cold” tumors. Its mechanisms involve defective antigen presentation, activation of immunosuppressive signaling, and impaired T-cell infiltration. Acquired resistance refers to disease progression following an initial response, accounting for over 60% of clinical treatment failures [18]. Key mechanisms include clonal expansion of immune escape variants (selective proliferation of low-immunogenicity tumor clones), upregulation of alternative checkpoint molecules, and epigenetic remodeling. Analysis of resistance mechanisms reveals that dysregulation of cell death pathways, particularly ICD, plays a pivotal role [17]. ICD influences key aspects of the TME, including infiltration of immunosuppressive cells, cytokine network balance, and the antigen-presenting capacity of DCs, through its unique “death-immunity interplay” mechanism. Obviously, a close functional interplay exists between ICD and immunotherapy [19]. Strategies such as combining ICD inducers with ICIs and targeted combination therapies modulating death pathways have demonstrated clinical potential in reversing resistance, offering novel therapeutic avenues to overcome immunotherapy resistance [20, 21]. Owing to their capacity to reshape the tumor microenvironment (TME) and inhibit tumor progression, both necroptosis and pyroptosis hold significant opportunities for cancer therapy. This review highlights recent advances in our understanding of pyroptosis and necroptosis, emphasizing their context-dependent roles in cancer progression and their therapeutic implications for overcoming cancer immunotherapy resistance and guiding precision cancer therapy strategies.

## Mechanistic insights into necroptosis and its immunotherapy potential

### Molecular mechanisms underlying necroptosis

**Fig. 1:**
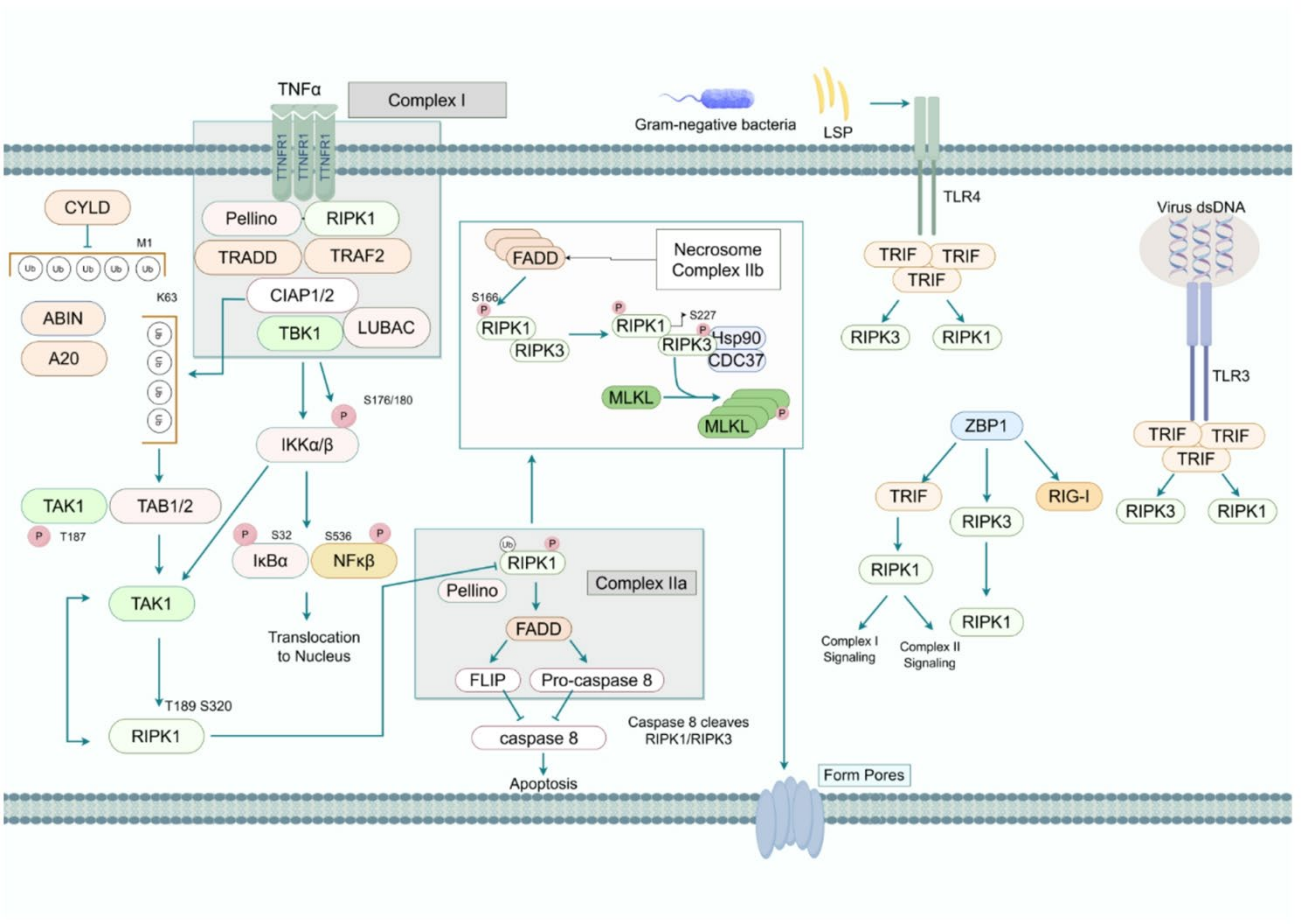
Mechanism of Activation and Signaling Pathways of Necroptosis TNFα induces TNFR1-CYLD-RIPK1 signaling to form the RIPK1-RIPK3 necrosome, activating MLKL and causing membrane rupture. TLR3/4 and ZBP1 recruit RIPK3-MLKL via RHIM to trigger necroptosis. Caspase-8 inactivation promotes, while Necrostatin-1 inhibits necroptosis

Necroptosis is a regulated form of PCD defined by unique morphological features, including cellular swelling, plasma membrane rupture, and the uncontrolled release of intracellular contents, thereby eliciting strong inflammatory responses. Unlike apoptosis, which involves apoptotic body formation, necroptosis directly disrupts the plasma membrane through pore formation, enabling release of cytoplasmic material [12, 14]. This process is tightly controlled through a phosphorylation cascade involving receptor-interacting serine/threonine-protein kinase 1(RIPK1), receptor-interacting serine/threonine-protein kinase 3 (RIPK3), and mixed-lineage kinase domain-like protein (MLKL), ultimately culminating in the assembly of the necrosome. Necroptosis can be activated by diverse stimuli, including death receptor signaling, pathogen infection, chemotherapeutic agents, or radiation exposure, and proceeds through two principal routes: death receptor-dependent and death receptor-independent pathways [11, 12, 14]. In the canonical death receptor-dependent pathway, as shown in Fig. 1, tumor necrosis factor-α (TNF-α) engages its cognate receptor TNFR1 at the plasma membrane, inducing receptor trimerization and subsequent recruitment of intracellular death domains to assemble Complex I, which contains TRADD, RIPK1, TRAF2/5, and cIAP1/2 [22]. Ubiquitination of RIPK1 by cIAP1/2 activates NF-κB and MAPK signaling, thereby promoting cell survival and preventing premature death signaling [23]. Under specific pathophysiological conditions, including oxidative stress or viral infection, the deubiquitinase CYLD removes K63-linked ubiquitin chains from RIPK1, leading to Complex I disassembly and transition into the cytosolic Complex IIa, which is composed of FADD, TRADD, procaspase-8, and deubiquitinated RIPK1. Its core function is to trigger the caspase-8-mediated proteolytic cascade, which subsequently induces apoptotic cell death [22]. In this setting, caspase-8 functions as a molecular switch in this regulatory network, where its enzymatic activity dictates the ultimate cell death modality. Inhibition of caspase-8 activity—whether by viral protease inhibitors or pharmacologic agents—shifts the signaling cascade toward necroptotic pathways. When caspase-8 is inactivated, deubiquitinated RIPK1 interacts specifically with RIPK3 via its RIP homotypic interaction motif (RHIM) domain to form a heterodimeric complex [13]. This molecular interaction triggers autophosphorylation of the RIPK3 kinase domain at Ser227, markedly enhancing its catalytic activity. The activated RIPK3 then recruits and phosphorylates its downstream effector MLKL, culminating in necrosome formation, a signaling complex that integrates these three key components [22]. Molecular dynamics studies have revealed that the N-terminal kinase domain of RIPK1 mediates RIPK3 phosphorylation, and the C-terminal death domain contributes to maintaining the structural stability of the complex. This modular architecture allows RIPK1 to precisely regulate signal intensity while preventing aberrant MLKL activation [13]. MLKL, as the terminal effector, undergoes conformational rearrangement upon dual phosphorylation at Thr357/Ser358. Following dissociation from the necrosome, phosphorylated MLKL oligomerizes via its N-terminal four-helix bundle domain, forming stable homotetramers. This oligomeric structure exposes C-terminal transmembrane α-helices, which specifically interact with and insert into cardiolipin-enriched membrane microdomains. Experimental evidence confirms that MLKL tetramers form transmembrane pores (~10nm) in the plasma membrane, causing ionic gradient disruption by driving Na^+^/Ca^2+^ influx and K^+^ efflux, leading to osmotic swelling and NLRP3 inflammasome activation, thus amplifying inflammation [13, 22]. The dynamic equilibrium of the classical pathway relies on multiple regulatory mechanisms. Under physiological conditions, homeostatic balance is maintained through opposing actions of cIAP1/2-mediated ubiquitination and CYLD-dependent deubiquitination, while Caspase-8-mediated cleavage of RIPK1/RIPK3 sustains signal quiescence. The small molecule inhibitor Necrostatin-1 selectively blocks necrosome formation by occupying the ATP-binding pocket of the RIPK1 kinase domain without affecting NF-κB signaling, representing a potential therapeutic target for ischemia-reperfusion injury. Notably, RIPK1 exhibits distinct functional roles across different complexes: within Complex I, it acts as a scaffolding protein to promote survival signaling, whereas in Complex IIb, it serves as a kinase to drive cell death execution [22, 23]. Beyond receptor-dependent pathways, necroptosis activation can also be triggered independently of death receptors through TLR3/4 and Z-DNA binding protein 1 (ZBP1) [24, 25]. Following viral dsRNA detection by TLR3 or bacterial LPS engagement with TLR4, TRIF directly recruits RIPK3 through RHIM domain interaction [24]. There are two distinctive features of this interaction. Firstly, it completely bypasses RIPK1-dependent regulation. Secondly, it specifically assembles TRIF-RIPK3-MLKL complexes when caspase-8 activity is suppressed. This interaction is characterized by two key features: bypassing RIPK1-dependent regulation and assembling TRIF–RIPK3–MLKL complexes under caspase-8 inhibition. Notably, TRIF-mediated necrosome formation exhibits spatiotemporal specificity, typically occurring after a pathogen breach of lysosomal barriers. This precise regulatory mechanism ensures immune efficacy while minimizing collateral damage to host tissues [26]. Recent studies reveal the indispensable role of ZBP1 in antiviral defense against enveloped viruses like herpesviruses, as it not only detects exogenous nucleic acids but also monitors endogenous Z-DNA generated during abnormal viral replication. ZBP1 activation occurs through binding of viral Z-DNA/Z-RNA, inducing direct RHIM domain-mediated interaction with RIPK3. This interaction subsequently triggers MLKL oligomerization and plasma membrane disruption [27, 28]. This surveillance mechanism significantly enhances cellular defense ability. Compared to the canonical RIPK1-dependent pathway, TLR pathways mediate broad-spectrum microbial defense, and ZBP1 specializes in nucleic acid virus clearance [25, 26]. Molecular dynamics simulations reveal distinct conformational requirements for RHIM-mediated interactions: TRIF-RIPK3 complex formation requires caspase inhibitor environments, whereas ZBP1 activation depends on IFN pre-stimulation [29]. This hierarchical regulation ensures immune response precision.

### Necroptosis at the crossroads of tumor Immunity and therapeutic resistance

The fundamental distinction between necroptosis and apoptosis lies in their different mechanisms of inflammatory response and immune modulation [11]. Necroptosis not only provokes pro-inflammatory responses, but also shapes anti-tumor immunity by releasing DAMPs that recruit immune cells into the TME and enhance their activation, thereby remodeling immune landscapes [30]. For example, F-actin released from necroptotic cells is sensed by Clec9A on DCs, activating Syk kinase signaling to enhance antigen uptake, processing, and presentation [31, 32]. Currently, phosphatidylserine exposure facilitates macrophage-driven efferocytosis via MFG-E8 and TIM4 receptors, facilitating the clearance of dying cells and secretion of anti-inflammatory factors [33]. Among DAMPs, HMGB1 plays a central role: it is released during necroptosis and stimulates NF-κB and MAPK signaling in DCs via TLR4 or RAGE receptors, upregulating co-stimulatory molecules CD80/CD86 and MHC class II expression [34]. HMGB1-TLR4 signaling has been found to elevate the maturation rate of DCs and significantly increase intratumoral CD8^+^ T-cell infiltration in melanoma [35, 36]. It also promotes macrophage secretion of TNF-α, IL-6, and IL-12, favoring M1 polarization [37, 38]. Similarly, ATP released during necroptosis binds P2X7 receptors, inducing potassium efflux and NLRP3 inflammasome activation. This amplifies IL-1β secretion, enhances DCs pro-inflammatory phenotypes, and potentiates macrophage inflammatory functions [39–41]. In addition, mitochondrial DNA (mtDNA) released during necroptosis activates the cGAS-STING pathway in macrophages, driving IFN-α/β production, enhancing antigen presentation, and promoting DCs migration to lymph nodes [42, 43]. Critically, RIPK3-activated DCs secrete IL-12, significantly enhancing the proliferation efficiency of CD8^+^ T-cells [44, 45]. In colorectal cancer (CRC), RIPK3 deficiency correlates with increased N2-type tumor-associated macrophages (TAMs) and enhanced tumor aggressiveness [46]. The activity of inflammatory cells, together with the type and expression level of inflammatory regulatory factors, precisely modulates the balance between pro- and anti-tumor effects. During acute inflammation, a moderate inflammatory response can suppress tumorigenesis through immune surveillance mechanisms. However, progression to chronic inflammation establishes a tumor-promoting microenvironment. Chronic necroptosis plays a key role in this process, where persistent release of inflammatory mediators significantly elevates local inflammation levels and promotes tumor progression. For instance, by releasing pro-angiogenic factors (e.g., VEGF, FGF) and matrix metalloproteinases, necroptosis stimulates pathological vascular proliferation and extracellular matrix degradation. These effects collectively foster tumor angiogenesis and enhance invasive potential, thereby facilitating tumor metastasis [30]. Sustained necroptotic activity induces MDSCs expansion and macrophage polarization toward an M2-like phenotype by secreting immunosuppressive molecules like S100A9, ultimately exacerbating immune evasion [30, 47]. For instance, MLKL-mediated necroptosis releases CXCL5, which recruits MDSCs and suppresses T-cell activity while elevated RIPK3 expression drives an immunosuppressive microenvironment through CXCL1, thereby accelerating tumor progression [48–50]. Thus, although necroptosis can initiate protective anti-tumor immunity, chronic necroptotic activity remodels the TME toward tumor growth, invasion, and cancer immunotherapy resistance.

## Mechanistic insights into pyroptosis and its immunotherapy potential

### Molecular mechanisms underlying pyroptosis

**Fig. 2:**
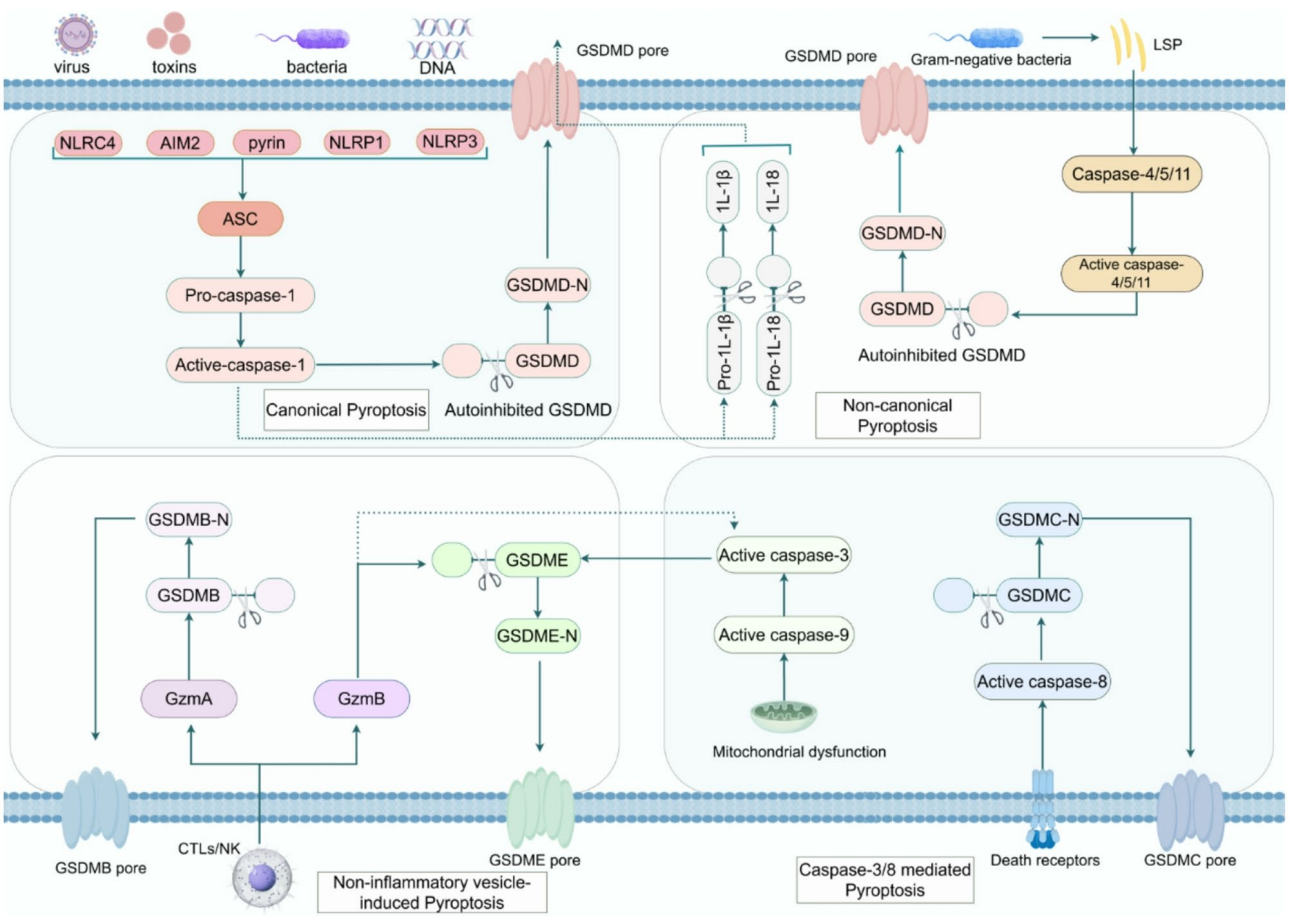
Mechanism of Activation and Signaling Pathways of Pyroptosis. PRRs activate ASC, triggering caspase-1 to cleave GSDMD, forming membrane pores and releasing IL-1β/IL-18. Non-canonical pyroptosis involves caspase-4/5/11 and GSDMD. Caspase-3/−8 or granzymes cleave GSDME, GSDMC, or GSDMB, inducing pyroptosis under specific stimuli

Pyroptosis is an inflammasome-dependent PCD distinct from other forms of cell death due to its strong association with inflammatory response and can be broadly classified into three pathways: canonical inflammasome, non-canonical inflammasome, and non-inflammatory vesicle-induced pathways (Fig. 2).

### Canonical pyroptosis

The canonical inflammasome signaling pathway was the first identified mechanism of the pyroptosis pathway. The core mechanism of the canonical inflammasome signaling pathway is the formation of inflammasomes, multiprotein complexes that integrate three core components: sensor proteins containing intracellular pattern recognition receptors (PRRs), apoptosis-associated speck-like proteins (ASCs) with both caspase activation and recruitment domains (CARD) and pyrin domains (PYD), along with the procaspase-1 zymogen precursor [51, 52]. Thus far, five inflammasomes—NLRP1, NLRP3, NLRC4, AIM2, and Pyrin—have been identified as mediators of pyroptosis, each defined by its distinct PRR. Upon activation of this pathway, PRR-containing sensor proteins selectively recognize pathogen-associated molecular patterns (PAMPs) or DAMPs. This recognition event triggers the recruitment of ASC, an adaptor protein that bridges sensor proteins to procaspase-1. In this complex, caspase-1 precursors are converted into active caspase-1 through proximity-induced self-cleavage. Active caspase-1 then cleaves GSDMD to produce GSDMD-C and GSDMD-N. The GSDMD-N fragment possesses intrinsic pore-forming activity, allowing it to penetrate cell membranes and form pores [53, 54]. Additionally, caspase-1 directly processes the precursors of the pro-inflammatory cytokines IL-1β and IL-18, converting them into their biologically active mature forms. These mature cytokines can then be released outside the cell through the pores formed by GSDMD in the cell membrane, thereby altering the local inflammatory milieu.

### Non-canonical pyroptosis

The noncanonical inflammasome pathway is mediated by caspase-4/5/11 [55, 56]. The CARD structural domains of caspase-4/5 and caspase-11 are so specific that they can directly recognize LPS from Gram-negative bacilli, and then undergo oligomerization and activation [57]. Similar to caspase-1, activated caspase-4/5/11 mediates pyroptosis by cleaving the substrate protein GSDMD [58]. However, the caspase-4/5 pathway is not isolated. The noncanonical inflammasome pathway can activate caspase-1, creating a positive feedback loop that further amplifies the inflammatory response [59]. In addition to caspase-1/4/5/11, other caspases have also been implicated in pyroptosis. Caspase-3 differs from caspase-1/4/5/11 in that it contains only one C-terminal caspase domain common to the caspase family, which hydrolyzes target proteins [60]. Moreover, caspase-3 lacks the capacity for autocatalysis or autocleavage. Thus, it was initially considered to play a role exclusively in non-inflammatory apoptosis, functioning as the terminal effector in the apoptotic caspase cascade. However, recent studies have shown that caspase-3, once activated by caspase-8 or related proteases, can directly cleave GSDME to initiate pyroptosis, expanding its role beyond apoptosis [61, 62]. Studies have demonstrated that multiple chemotherapeutic agents, including lobaplatin, doxorubicin, and apoptin, can activate caspase-3, thereby cleaving GSDME and inducing pyroptosis [63]. Targeting GSDME to modulate the pyroptosis response is an extremely promising pathway in tumor therapy. Another caspase that can induce pyroptosis is caspase-8 [64]. It can induce GSDME-mediated pyroptosis indirectly by regulating caspase-3 or directly trigger pyroptosis by cleaving GSDMC [64]. In the presence of caspase-1, ZBP1-RIPK3 scaffold recruits caspase-8 and facilitates NLRP3 inflammasome-dependent activation, ultimately triggering pyroptotic cell death [65]. Alternatively, under acidic conditions, α-ketoglutarate metabolism generates elevated reactive oxygen species (ROS) that promote death receptor 6 internalization from the plasma membrane. This process facilitates the recruitment of procaspase-8 and GSDMC. Caspase-8-mediated cleavage of GSDMC generates an N-terminal fragment, by analogy to GSDMD, that forms plasma membrane pores and induces pyroptotic cell death [66].

### Non-inflammatory vesicle-induced pyroptosis

In parallel to the above pathways, granzymes secreted by CTLs and NK cells directly cleave GSDM family proteins. Specifically, granzyme B cleaves GSDME at its interdomain linker region, liberating the N-terminal fragment that executes pyroptosis [67, 68]. Granzyme B not only directly cleaves the GSDM protein but also cleaves and activates caspase-3. Caspase-3 further cleaves the GSDME and amplifies the pyroptosis signal. In AML patients, the overexpression of CD155 on tumor cells promotes the endocytosis and degradation of DNAM-1 on ILC2s, leading to impaired GZMB secretion and cancer immune evasion [69]. Granzyme A induces pyroptosis by recognizing and cleaving a specific site on GSDMB, releasing its N-terminal domain, which then forms a membrane pore. Both anti-CD19 CAR-T cells and NY-ESO-1-specific TCR-T cells induce pyroptosis in target cells via GZMA-mediated cleavage of GSDMB, an effect dependent on GSDMB integrity. Thus, modulating GZMA expression in CAR-T/TCR-T cells or combining them with GSDMB agonists may enhance their immunotherapy efficacy against solid tumors [70]. Collectively, granzyme-mediated pyroptosis represents a caspase-independent pathway that integrates fundamental cytotoxic mechanisms with inflammatory cell death, ensuring both rapid immune response and autonomous pyroptosis activation.

### The emerging role of pyroptosis in cancer immunotherapy resistance

**Fig. 3:**
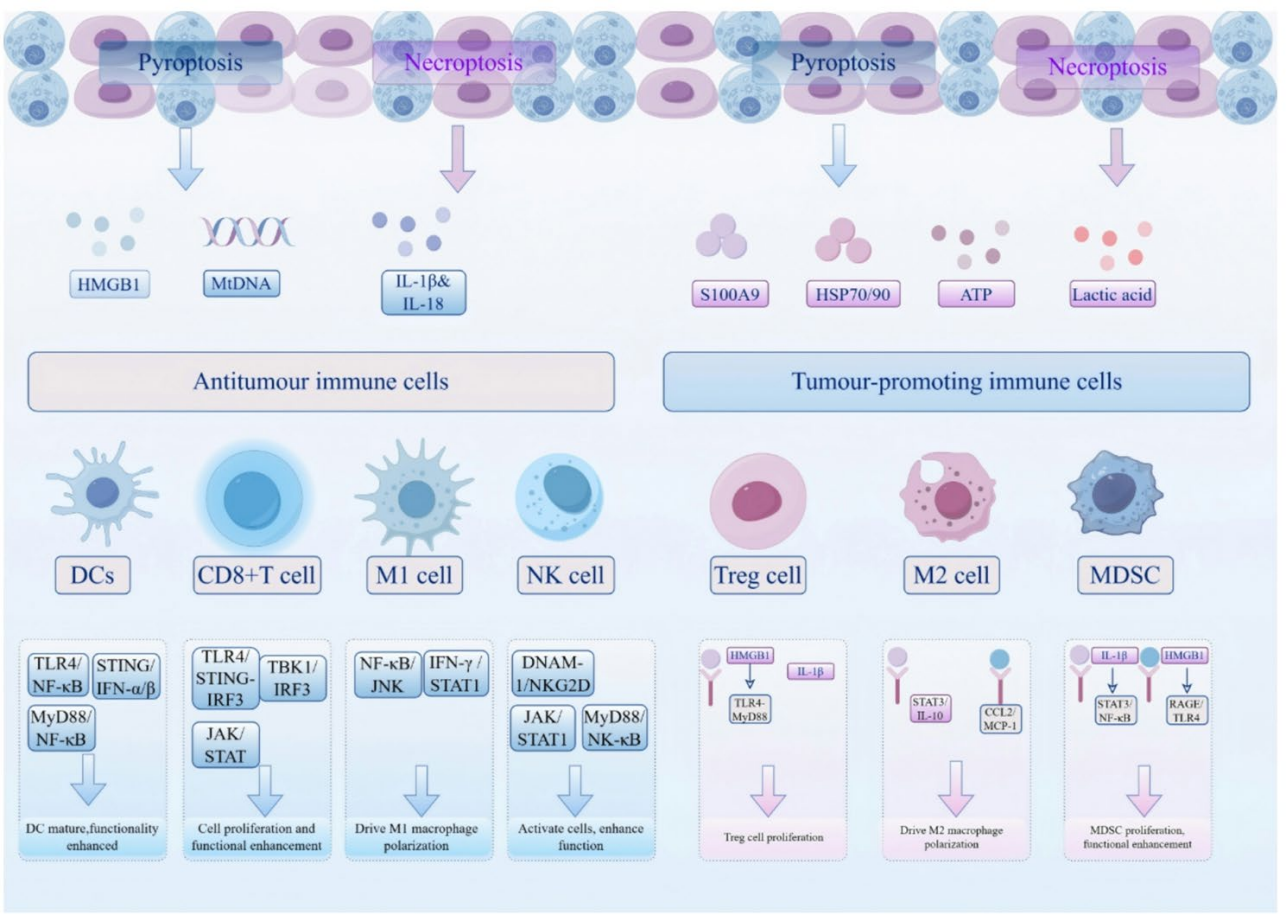
Schematic illustration of the effects of pyroptosis and necroptosis on reshaping the TME. Pyroptosis and necroptosis release mediators like HMGB1, mtDNA, IL-1β, IL-18, S100A9, HSP70/90, ATP, and lactate, which shape immune responses depending on the microenvironment. These signals can activate anti-tumor immunity by enhancing DC maturation, CD8^+^ T cells, M1 macrophages, and NK cells, but may also promote tumor-supportive immunity by inducing Tregs, M2 macrophages, and MDSCs

Pyroptosis, akin to necroptosis, serves as a critical nexus between cell death and cancer immunotherapy within the TME, exerting a remarkable effect on tumor progression [71, 72]. While it can suppress tumor growth by initiating antitumor immune responses, inducing tumor cell lysis, and limiting metastasis, persistent activation may conversely promote tumor progression through chronic inflammation and TME remodeling [71, 73]. The biological effects of pyroptosis are highly context-dependent, varying by cancer types, cellular origin, treatment regimen, and the duration of inflammation. In terms of antitumor activity, pyroptosis triggers the release of DAMPs, such as HMGB1 and mtDNA, which promote DCs maturation and antigen presentation, thereby enhancing CD8^+^ T cell infiltration and adaptive immune responses (Fig. 3). GSDME-mediated pyroptosis significantly increases tumor infiltration of CD8^+^ T cells by releasing IL-1β and IL-18, effectively reprogramming the immunosuppressive TME [74]. Directly mediating tumor cell death is a fundamental mechanism by which pyroptosis overcomes cancer treatment resistance. Chemotherapeutic agents such as oxaliplatin or doxorubicin induce pyroptosis via caspase-3-mediated GSDME cleavage—an effect further potentiated by farnesoid X receptor (FXR) agonists like GW4064 [75]. In addition, pyroptosis remodels the immunosuppressive TME by inhibiting the production of MDSCs and M2-like macrophages, while promoting macrophage polarization to M1 through IL-1β and IL-18 release. Moreover, it restrains metastasis by enhancing CD8^+^ T cell infiltration, inhibiting epithelial-mesenchymal transition (EMT) signaling pathway, and suppressing tumor cell motility [73]. However, similar to necroptosis, chronic inflammation driven by pyroptosis can promote tumor progression. Molecules released during persistent pyroptosis, such as ROS and IL-1β, induce DNA damage (e.g., APC gene mutations in CRC) and accelerate adenoma-to-carcinoma transformation. Metabolites released during pyroptosis, such as ATP and lactate, drive macrophage polarization toward the M2 phenotype and enhance secretion of pro-angiogenic factors like VEGF, thereby creating a tumor-supportive microenvironment that facilitates metastasis [76]. Interestingly, pyroptosis plays a highly heterogeneous role in cancer treatment resistance across different tumor types [71, 73]. Given this duality, regulating pyroptosis has emerged as a promising strategy in cancer therapy. Current research focuses on harnessing its synergy with ICIs and targeted therapies to overcome resistance in advanced tumors. Ongoing clinical trials and nanomedicine-based delivery systems are expected to accelerate the translation of pyroptosis-based interventions into the clinic.

## Necroptosis and pyroptosis in immunotherapy resistance across various cancer types

This review summarizes recently identified molecules capable of modulating necroptosis and pyroptosis for therapeutic benefit across diverse cancers (Table 1). More importantly, we identified potential therapeutic targets within these pathways that hold significant promise for the development of novel anticancer therapies (Table 2).

**Table 1:** Novel compounds inducing necroptosis or pyroptosis with promising anticancer potential

Promising therapeutic drugs or molecules	ICD subtypes	Pathways	Tumor types	Reffence
HHT	necroptosis	enhance MLKL inactivation	CRC	78
Fructose	necroptosis	affect the glycolysis pathway	CRC	89
pOEG-b-D-SH@NP	necroptosis	induce CT26 CRC cell necroptosis	CRC	80
OSW-1	necroptosis	RIPK1-p62/SQSTM1 complex	CRC	82
Buffalo Milk Whey	necroptosis	activate the RIPK1, RIPK3 and MLKL	CRC	83
Kushen Injection	necroptosis	enhance the necroptosis of SW480 cells	CRC	84
MicroRNA-29b-3p	necroptosis	suppress TRAF5-mediated necroptosis	CRC	85
GW4064	pyroptosis	enhance pyroptosis mediated by GSDME	CRC	75
CaZCH NPs	pyroptosis	induce mitochondrial calcium overload	CRC	96
FL118	pyroptosis	activate the NLRP3 inflammasome	CRC	224
GSK0547	necroptosis	highly selective and effective RIP1 inhibitor	PC	101
BV6	necroptosis	induce necroptosis in apoptosis-resistant pancreatic cancer cells	PC	104
HSA NPs	pyroptosis	target GLUT1 and ASCT2	PC	111
Pt-In NPs	pyroptosis	enhance platinum colocalization	PC	109
BIX-01294	necroptosis	inhibit G9a histone methyltransferase activity	BC	120
organoantimony(III)	necroptosis	induce necroptosis	BC	126
ORIN1001	pyroptosis	restore dsRNA accumulation and pyroptosis signal transduction	BC	128
Click-APP-DOX	pyroptosis	induce GSDME-mediated pyroptosis	BC	130
DHA	pyroptosis	activate caspase-1 and induce pyroptosis	BC	223
T22-PE24	pyroptosis	induce GSDME-mediated pyroptosis	HCC	148
MF@SOR	pyroptosis	induce pyroptosis	HCC	149
cannabidiol	pyroptosis	induce GSDME-mediated pyroptosis	HCC	151
reuteri	pyroptosis	enhance mtDNA-mediated STING activation and caspase 8 expression	HCC	152
Tolinapant	necroptosis	behave as an inhibitor of apoptosis protein (cIAP1, cIAP2, and XIAP)	TCL	157
CN-Pt-GEM	necroptosis	induce necroptosis	NSCLC	168
TC14012	necroptosis	inhibit endothelial cell necroptosis	LC	169
HCNP	pyroptosis	induce GSDME-mediated pyroptosis	LC	175
Cas-CMV@LM	necroptosis	induce necroptosis in leukemia primitive cells	AML​	178
EG2	pyroptosis	induce AML cells to differentiate into DCs	AML	181
ANA&IDA	pyroptosis	induce GSDME-mediated pyroptosis	AML	182
5-FU@HFn	pyroptosis	induce GSDME-mediated pyroptosis	CML	183
CLQ&VEN	pyroptosis	induce pyroptosis through IFIT1/IFIT3	AML​	184
CBL0137	necroptosis	activate ZBP1-dependent necroptosis	MM	186
3u	necroptosis	induce necroptosis	MM	187
oregano	necroptosis	induce necroptosis	MM	188
AZD1775	necroptosis	activate BATF3-dependent DCs	MM	189
TPL@TFBF	pyroptosis	increase ROS level	MM	191
recombinant human adenovirus 5	pyroptosis	induce pyroptosis	MM	192
TREM2	pyroptosis	induce GSDME-mediated pyroptosis	MM	193
terphenyllin	pyroptosis	induce pyroptosis	MM	194
C8	necroptosis	induce necroptosis	GC	203
IBI315	pyroptosis	cleavage GSDMB	GC	205
APG-1252	pyroptosis	block phosphorylation of AKT/GSK-3β/MCL-1	GC	206
7I	necroptosis	Activate P53/P21/CDK1/cyclin B pathway	GBM	211
IL-12 mRNA@cRGD-CM-CaCO₃NPs	necroptosis	induce necroptosis and stimulate CTL proliferation	GBM	212
CuCCT@CM-Ang	pyroptosis	penetrate the blood-brain barrier and precisely induce pyroptosis	GBM	215
OTA NPs	pyroptosis	induce mtDNA release and activate the AIM2/ASC-dependent pathway	GBM	217
IASNDS	pyroptosis	lead GBM cell membrane perforation and cytoplasmic leakage	GBM	218

**Table 2:** Identified potential therapeutic targets mediated by necroptosis and pyroptosis across various tumor types

Promising therapeutic target	ICD subtypes	Pathways	Tumor types	Reffence
TRAF6	necroptosis	degrade RIPK1	CRC	81
IL-17	pyroptosis	increase infiltration of CD8 + T cells	CRC	74
β5-integrin	pyroptosis	over-activate the Src-STAT3-ASAH2 pathway	PADC	108
G9a histone methyltransferase	necroptosis	activate the TNF–p53–RIPK3–MLKL pathway	BC	120
AQP1	necroptosis	interact directly with the D324 residue of RIPK1	BC	122
LT-IIc	necroptosis	induce necroptosis in TNBC cells	BC	123
BAD	necroptosis	inhibit cyclin B1 and increase the generation of ROS	BC	124
YARS	necroptosis	induce necroptosis	BC	125
IRE1 α	pyroptosis	degrade dsRNA	BC	128
CHMP3	pyroptosis	inhibit Caspase-1-dependent pyroptosis	HCC	153
pir-has-216,911	pyroptosis	block the TLR4/NFκB/NLRP3 signaling pathway	HCC	154
NEK7	pyroptosis	Knockdown of NEK7 inhibits the activation of hepatic stellate cells and cancer-interstitial interactions	HCC	155
TLR4	pyroptosis	upregulate TLR4 expression in PTCL cells	PTCL	162
SAMHD1	pyroptosis	inhibit STING activation and pyroptosis induced by DNA damage	DLBCL	164
SOX11	pyroptosis	Endogenous SAMHD1 inhibitor	MCL	165
miR-10b-5p	necroptosis	directly target PKP3 and inhibit RIPK3/MLKL necroptosis pathway	LUAD	170
Skp2	necroptosis	Skp2-mediate MLKL degradation	NSCLC	171
Smurf2	pyroptosis	reduce NLRP3 ubiquitination and increase its stability	NSCLC	172
circRPPH1		enhance SIRT1 gene transcription	LUAD	173
TNFAIP3/A20	necroptosis	target RIPK1, resulting in resistance of AML cells to anthracyclines	AML	176
R-2HG	necroptosis	upregulate RIPK1	AML	177
FLOT1	pyroptosis	inhibit pyroptosis	AML	180
PDE3A	pyroptosis	inhibit GSDME-mediated pyroptosis	AML	182
Hs02	pyroptosis	regulate pyroptosis-related component expression	AML	185
ADAR1	necroptosis	play a key role in resistance to ICB therapy	MM	186
hBMSC-Exos	pyroptosis	alleviate PD-1/PD-L1 inhibitor-induced myocardial injury	MM	198
miR-211-5p	pyroptosis	regulate glucose metabolism and pyroptosis through GNA15	MM	199
12-PRlncRNA	pyroptosis	predict prognosis and indicate tumor immune microenvironment in skin cutaneous melanoma	MM	200
MYOSLID	necroptosis	inhibit necroptosis	GC	202
ALKBH4	pyroptosis	reduce necroptosis and decrease chemosensitivity	GC	207
BRD2	pyroptosis	affect NF-κB/METTL3/BRD2/FOXO4-FLIP/Caspase-8 pathway	GC	208
TAK1	necroptosis	activate the Caspase-8/FADD complex	GBM	213
CSRP2	necroptosis	activate JAK-STAT1 signaling pathway	GBM	214
GOLPH3L	pyroptosis	bind to STING, and inhibits STING activation, thereby reducing pyroptosis	GBM	216

### CRC

In CRC, necroptosis serves as a critical PCD mechanism that plays a pivotal role in suppressing tumor initiation and progression. RIPK3 deficiency promotes the accumulation, proliferation, and immunosuppressive function of MDSCs, thereby accelerating colorectal carcinogenesis [77]. Homoharringtonine (HHT) synergizes with MLKL inactivation to suppress CRC cell growth, an effect not observed with other targeted agents. Targeting MLKL in combination with HHT treatment represents a promising therapeutic strategy for CRC [78]. Emerging evidence indicates that fructose suppresses hypoxia-induced necroptosis in human CRC cells through glycolytic modulation [79]. Additionally, a novel nanomedicine platform (pOEG-b-D-SH@NP) selectively targets CT26 CRC cells and effectively induces necroptotic cell death through the simultaneous disruption of the energy pathway, thereby countering the metabolic adaptability that limits current cancer therapies [80]. TRAF6 directly ubiquitinates and degrades RIPK1, inhibiting both necroptosis and apoptosis, thereby promoting cancer cell proliferation and tumor progression [81]. Furthermore, several compounds, including OSW-1, Buffalo Milk Whey, Kushen Injection, and MicroRNA-29b-3p, have recently been identified to exert anti-tumor effects by inducing necroptosis [82–85]. Although the contribution of pyroptosis to the development of CRC along the normal mucosa–adenoma–carcinoma sequence remains incompletely defined, accumulating evidence suggests that pyroptosis exerts a critical inhibitory effect on tumor progression [86]. NLRP3 inflammasomes protect colonic mucosa during acute colitis, and their deficiency exacerbates chemically induced colitis-associated CRC [87, 88]. Similarly, *Nlrc4*-deficient mice exhibit heightened sensitivity to dextran sulfate sodium (DSS)-induced colitis compared to wild-type controls [89]. However, it has also been found that the occurrence of pyroptosis may contribute to the growth of CRC under specific circumstances, especially during chronic inflammation of colitis. For example, in chronic colitis, IL-1β and ROS released during epithelial cell pyroptosis induce DNA damage, which in turn promotes mutations in the APC gene and accelerates colorectal carcinogenesis [76]. Persistent pyroptosis leads to sustained activation of NLRP3 inflammasomes, driving IL-1β overproduction, angiogenesis, and stromal remodeling, thereby creating an ecological niche for tumor metastasis [90]. Notably, high GSDMB expression in HER2-positive CRC is associated with chemotherapy resistance and metastasis [91]. Multiple therapeutic modalities have been developed in CRC. Pyroptosis-driven activation of the immune response can effectively overcome chemoresistance [92]. The combination of FXR agonist GW4064 and oxaliplatin synergistically inhibited CRC cell growth through GSDME-mediated pyroptosis in CRC cells [75]. LPS triggers pyroptosis to enhance the chemosensitivity of oxaliplatin on HT29 cells by promoting GSDMD expression [93]. Conversely, the elevated abundance of Fusobacterium nucleatum in CRC patients who relapse after chemotherapy inhibits the GSDME-mediated pyroptosis pathway, increasing resistance to 5-fluorouracil and oxaliplatin [94]. Although immunotherapy is currently limited to CRC patients with high microsatellite instability or deficient mismatch repair (representing approximately 15% of cases), the combination of pyroptosis induction with immunotherapy presents a promising therapeutic breakthrough for microsatellite-stable CRC patients [95]. Through its immunostimulatory effects, pyroptosis can transform immunologically “cold” tumors into “hot” tumors, thereby rendering them susceptible to immunotherapy. IL-17 significantly increases the infiltration of CD8^+^ T cells in TME, and is a potential target that can be used to enhance the efficacy of immunotherapy [74]. Calcium-based nanoinducers (CaZCH NPs) induce mitochondrial calcium overload and trigger pyroptosis, polarizing TAMs toward the M1 phenotype and alleviating immunosuppression [96]. These groundbreaking studies underscore the immense potential of pyroptosis in CRC-targeted therapy.

### Pancreatic cancer

Pancreatic ductal adenocarcinoma (PDAC) is classically regarded as an immunoindolent tumor, characterized by profound local immunosuppression. This is evidenced by its abundant immunosuppressive cell populations and striking deficiency of immunogenic effector cells, especially CD8^+^ CTLs [97]. Necroptosis acts as a multifaceted mechanism in PDAC. Accumulating evidence suggests that the pro-tumorigenic effects of necroptosis predominate in this malignancy [48, 98–100]. In pancreatic cancer, in vivo experiments reveal that RIPK1/3-mediated signaling increases the accumulation of MDSCs and TAMs by promoting the high expression of CXCL1 and SAP130, which contribute to establishing an immunosuppressive microenvironment [48–50]. Moreover, RIPK1 drives TAM polarization toward the M2-like phenotype by inhibiting STAT1 signaling. This leads to suppressed the activation of Th1, Th17, and CD8^+^ T cells, and thereby reinforcing systemic immune evasion [101]. Overexpression of MLKL upregulates CD47 expression via IL-6 signaling, which consequently suppresses macrophage phagocytosis and enhances tumor immune evasion. During the process of necroptosis, CXCL8 will be released. Through CXCL8/CXCR pathway activation, it induces macrophage extracellular trap (MET) formation, subsequently promoting extracellular matrix degradation and triggering the EMT program. These effects enhance tumor cell-endothelial adhesion and promote hepatic metastasis in PDAC [100]. These findings suggest that combinatorial strategies targeting MLKL and CD47 may offer novel neoadjuvant therapeutic opportunities for patients with early-stage resectable or borderline resectable disease, potentially restoring eligibility for surgical intervention. Additionally, MLKL expression levels and MET formation dynamics may serve as predictive biomarkers for liver metastasis in PDAC [100, 102, 103]. Building on these insights into the tumor immune microenvironment, the selective RIPK1 inhibitor GSK0547 has shown significant antitumor efficacy in preclinical models. This effect is mediated through the promotion of M1 macrophage polarization and the enhancement of infiltration and activation of Th1, Th17, and CD8^+^ T cells [101]. The Smac mimetic BV6 has been shown to synergize with the STING agonist cGAMP in inducing necroptosis in apoptosis-resistant pancreatic cancer cells. This combination therapy strongly activated NF-κB and IRF1 signaling pathways, significantly increasing cell death rates [104].

In PDAC, pyroptosis predominantly drives tumor suppression, unlike necroptosis. However, the influence of pyroptosis can swing to promote progression in particular niches of the TME [71, 105–107]. Several therapeutic strategies aimed at modulating pyroptosis are currently under investigation. Overexpression of β5-integrin has been reported to attenuate chemotherapy-induced pyroptosis in pancreatic cancer by activating the Src–STAT3–ASAH2 pathway, thereby promoting chemoresistance and contributing to unfavorable patient outcomes [108]. Self-assembled nanoparticles Pt-In NPs enhance platinum colocalization within tumor cells. It suppresses COX-2 expression to reduce prostaglandin E2 (PGE2) secretion, thereby inhibiting MDSCs recruitment. Concurrently, platinum-induced DNA damage activates the caspase-3/GSDME pyroptotic pathway which reshapes the immunosuppressive TME. Exposure of calreticulin and release of HMGB1 further amplify antigen presentation, and when combined with anti-PD-L1 therapy, this approach completely abolishes PDAC metastasis in preclinical models, effectively converting the “cold” tumor phenotype into a “hot” immune-inflamed state, thus opening new avenues for immunotherapy [109]. Src inhibitors (e.g., dasatinib) and albumin-bound nanoparticles (HSA NPs) demonstrate similarly significant therapeutic potential [110, 111]. In addition, photothermal therapy potentiates the activation of pyroptosis and anti-tumor immunity in PDAC when combined with a STING agonist and dronedarone hydrochloride [112, 113]. Given that elevated expression of gasdermins such as GSDMC, GSDME, or GSDMD has been implicated in the development of chemoresistance in PDAC, targeted modulation of these effectors presents another critical therapeutic direction for improving clinical outcomes in this disease [114–116].

### Breast cancer

Different from other tumors in which necroptosis is triggered through the typical RIPK1-RIPK3-MLKL pathway, breast cancer (BC) uniquely depends on ZBP1 as the central regulator of this process. Its deletion leads to a reduction of more than 50% in tumor necrotic area and a significant decrease in MLKL phosphorylation [28]. One strategy for immune evasion by primary tumors involves the epigenetic silencing of RIPK3 to avoid necroptosis [117, 118]. By contrast, recurrent BC epigenetically reactivate RIPK3 to drive proliferation and sustain tumor fitness via pathways including YAP/TAZ activation. This paradox illustrates a tumor adaptive strategy: sacrificing immune vulnerability for growth advantage [119]. G9a orchestrates immune evasion by silencing the expression of TNF through H3K9 dimethylation at gene promoters. Experimental evidence demonstrates that the G9a inhibitor BIX-01294 significantly reactivates this pro-inflammatory gene, subsequently activating p53 and triggering necroptosis [120]. Among BC molecular subtypes, triple-negative BC (TNBC) represents the most clinically challenging BC subtype, exhibiting strong biological aggressiveness, limited therapeutic vulnerabilities, and poor prognosis [121]. Mechanistically, aquaporin 1 (AQP1) has been identified as a key driver of TNBC progression, acting through direct interaction with the D324 residue of RIPK1 to inhibit necroptosis and downstream signaling. This interaction attenuates the release of DAMPs and inflammatory cytokines, enabling TNBC cells to disengage from immune surveillance. In vivo studies further demonstrate that either overexpression of RIPK1 or the D324K mutation significantly attenuates tumor progression and prolongs survival [122]. The bacterial type II heat-labile enterotoxin (LT-IIc) induces both apoptosis and necroptosis in TNBC cells by activating autophagy while simultaneously blocking the fusion of autophagosomes with lysosomes. This mechanism of LT-IIc leads to selective cytotoxicity against TNBC cell lines [123]. In addition, molecules such as the Bcl-2-associated death promoter (BAD), tyrosine aminoacyl-tRNA synthetase (YARS), and organoantimony(III) enhance the efficacy of conventional chemotherapeutic agents by inducing the necroptotic pathway. These findings offer promising avenues for combinatorial strategies in BC therapy [124–126]. The absence of estrogen receptor (ER), progesterone receptor (PR), and human epidermal growth factor receptor 2 (HER2) expression in TNBC significantly restricts available targeted therapeutic approaches [127]. Fortunately, pyroptosis-based therapeutic options have opened up new horizons for TNBC. Recent studies reveal a mechanism of immune evasion in TNBC mediated by IRE1α. Specifically, IRE1α utilizes its RNase activity to degrade dsRNA generated during paclitaxel treatment, thereby suppressing NLRP3 inflammasome activation and GSDMD-mediated pyroptosis. The IRE1α inhibitor ORIN1001 reverses the above mechanism, transforming PD-L1-negative “cold tumors” into PD-L1-positive “hot tumors”, significantly improving the reactivity to PD-1 inhibitors and immunogenicity of paclitaxel [128]. A bionic nanocrystal MG@PM consisting of mitoxantrone (MIT) and garcinol (GA) was designed to induce ribosomal stress and ROS accumulation to trigger pyroptosis [129]. Similarly, the Click-APP-DOX system achieved an 86.12% tumor inhibition rate, significantly outperforming free doxorubicin (50%), further validating the efficacy of pyroptosis-inducing strategies [130]. Additional studies have identified mitochondrial uncoupling protein 1 (UCP1), nigericin, and arsenic trioxide as effective inducers of pyroptosis in TNBC [131–133]. Beyond TNBC, pyroptosis has shown broad therapeutic relevance across other molecular subtypes of BC, with accumulating evidence supporting its translational potential [134–137].

### Liver cancer

Primary liver cancer encompasses two major histological subtypes—hepatocellular carcinoma (HCC) and intrahepatic cholangiocarcinoma (ICC)—which exhibit distinct pathological features, metastatic potentials, and therapeutic responses. ICC demonstrates more pronounced necroptotic signatures compared to HCC. Necroptosis in hepatocytes triggers the release of DAMPs, which activate immune cells to secrete diverse cytokine genes, including *Ccl4*, *Aimp1*, *Cxcl13*, *Ccl6*, *Ccl8*, *Pf4*, and *Osm*. During this process, a unique TME could be established that regulates chromatin accessibility and expression levels of lineage-determining transcription factors *Tbx3* and *Prdm5* through epigenetic mechanisms, thereby driving tumor differentiation toward the ICC lineage [138, 139]. Clarifying the roles of the liver microenvironment and key regulatory factors in HCC lineage targeting can help developing therapeutic strategies. Necroptosis dysregulation is intimately linked with hepatic pathogenesis. Upon LPS/TNF stimulation, RIPK1 stabilizes TRAF2 to modulate cell death pathways and inflammatory signaling to attenuate hepatic inflammation, hepatocyte injury, fibrosis progression, and carcinogenesis [140–142]. Conversely, low expression of RIPK3 in the necroptosis pathway leads to sublethal necrosome activation when NF-κB is activated, resulting in a “sublethal” cellular state that releases chemokines and promotes inflammatory carcinogenesis. Inhibiting NF-κB under these conditions would induce lethal necroptosis, restricting DAMPs release and preventing both inflammation and hepatocarcinogenesis. This regulatory mechanism positions NF-κB as a molecular switch governing divergent cell death outcomes. Clinicopathological analyses of non-alcoholic fatty liver disease biopsies have identified co-activated NF-κB and necroptotic signaling regions, with patients exhibiting distinct transcriptional profiles demonstrating poorer clinical outcomes. This finding suggests that pharmacological modulation rather than direct inhibition of necroptotic pathways may represent an effective therapeutic strategy for liver cancer treatment [143, 144]. Due to the critical role of necroptosis in the development of HCC, therapeutics based on it hold great promise, such as cisplatin, sunitinib, and sanguinarine, which have demonstrated the potential to achieve anti-tumor effects by inducing necroptosis [145, 146].

Researchers have developed a pyroptosis-associated gene-based risk stratification system that characterizes pyroptosis-related immune microenvironments, providing critical insights for HCC immunotherapy [147]. CXCR4-targeted self-assembling cytotoxic nanotoxin T22-PE24 and smart responsive bimetallic nano-vaccine MF@SOR demonstrate effective pyroptosis induction in HCC cells, promoting DCs maturation and immune microenvironment remodeling with considerable tumor inhibition rates at primary and metastatic sites, respectively [148, 149]. Porphyrin-conjugated mesoporous silica nanoparticle-mediated sonodynamic therapy combined with anti-PD-L1 blockade enhances therapeutic efficacy by inducing pyroptosis [150]. Other modalities, including cannabidiol and lactobacillus reuteri metabolite reuterin, have demonstrated anti-hepatocarcinogenic effects [151, 152]. Ongoing targets like CHMP3, pir-hsa-216911, and NIMA-related kinase 7 (NEK7) continue to expand the therapeutic landscape of pyroptosis in liver cancer treatment [153–155]. Thus, these insights highlight that targeted modulation of necroptosis and pyroptosis represents a promising immunotherapeutic strategy to overcome immune evasion and enhance anti-tumor immunity in HCC.

### Lymphomas

Thymoquinone exhibits potent cytotoxicity against diffuse large B-cell lymphoma (DLBCL), with superior antitumor effects compared to conventional chemotherapeutic agents while sparing normal hematopoietic cells. Its primary mechanism involves induction of necroptosis, suggesting that necroptosis-based interventions may represent a promising strategy for overcoming drug resistance in DLBCL [156]. Tolinapant, a selective non-peptidomimetic inhibitor of cellular IAPs (cIAP1, cIAP2, and XIAP), has emerged as a promising therapeutic agent for T-cell lymphomas [157]. CRISPR-based mechanistic studies of necrosome components RIPK3 and MLKL in T-cell lymphoma models demonstrate that loss of these proteins substantially impairs tumor cell response to toltrazuril. Importantly, decitabine enhances toltrazuril-induced necroptosis by reversing epigenetic suppression of RIPK3 and MLKL through DNA demethylation. Both of these two drugs have shown preclinical immunomodulatory anti-cancer potential, providing the basic principle for combined treatment [158–160]. Furthermore, trabectedin has shown therapeutic promise in overcoming doxorubicin resistance by inducing necroptosis-mediated modulation of TME cell recruitment and monocyte immunosuppressive polarization, particularly in classical Hodgkin lymphoma [161].

In peripheral T-cell lymphoma (PTCL), stromal macrophages undergo pyroptosis, characterized by IL-18 secretion. IL-18 upregulates TLR4 expression in PTCL cells, activating the downstream NF-κB-mediated anti-apoptotic pathway to promote tumor proliferation and chemoresistance. TLR4 knockdown reverses this pathogenic cascade, establishing TLR4 as a potential therapeutic target in PTCL [162]. The Chinese-developed histone deacetylase (HDAC) inhibitor chidamide has been demonstrated to exert antitumor effects through caspase-3 activation. In T-cell lymphoblastic lymphoma/leukemia (T-LBL/ALL), HDAC1, HDAC2, and HDAC3 are all aberrantly overexpressed, presenting potential therapeutic targets [163]. SAMHD1 is highly expressed in DLBCL and may be a promising therapeutic target due to its ability to suppress DNA damage-induced STING activation and thereby block pyroptosis. Combination therapy with the STING agonist DMXAA and PD-1 inhibitors demonstrates synergistic antitumor activity against DLBCL [164]. Additionally, the endogenous SAMHD1 inhibitor SOX11 has been identified as a therapeutic target in mantle cell lymphoma (MCL) [165].

### Lung cancer

In non-small cell lung cancer (NSCLC), reduced expression of RIPK3 and MLKL is significantly associated with poor patient prognosis, suggesting that pathway inactivation may promote tumor immune evasion. Inhibitors targeting RIPK1 (like nec-1) overcome partial chemotherapy resistance, implying that the necroptosis pathway represents a promising therapeutic target in lung cancer [166, 167]. The investigation of necroptosis in NSCLC has expanded beyond conventional targeted therapies or combinatorial approaches involving immunotherapy and chemotherapy. Researchers designed a composite hydrogel called CN-Pt-GEM, and it could leverage the photothermal conversion properties of single-atom platinum and gemcitabine to induce necroptosis in residual cancer cells through near-infrared light activation. This therapeutic strategy not only upregulates DAMPs but also drives macrophage polarization toward the M1 phenotype. When applied to surgical wound surfaces, this hydrogel effectively inhibits postoperative NSCLC recurrence by modulating the TME, thereby establishing a novel therapeutic paradigm for addressing tumor relapse after surgical resection [168]. Regarding tumor metastasis, emerging evidence reveals that the CXCR7 agonist TC14012 exerts anti-metastatic effects by suppressing endothelial cell necroptosis, thereby inhibiting transendothelial migration of tumor cells and lung cancer metastasis. It is important that this effect is mediated specifically through CXCR7 rather than its closely related receptor, CXCR4, identifying CXCR7 as a potential therapeutic target for lung cancer treatment [169]. The expression of miR-10b-5p is significantly elevated in lung adenocarcinoma (LUAD) tissues, where it promotes cancer progression by directly targeting plakophilin 3 (PKP3) and suppressing the RIPK3/MLKL necroptosis signaling pathway. Targeting miR-10b-5p may be a novel therapeutic strategy [170]. Furthermore, the E3 ubiquitin ligase SKP2 is overexpressed in NSCLC cell lines, displaying an inverse correlation with MLKL protein levels. Targeting the Skp2-mediated ubiquitination-degradation pathway of MLKL reverses cisplatin resistance in NSCLC [171].

Regarding pyroptosis, emerging research highlights the regulatory role of the ubiquitin ligase SMURF2 in NSCLC progression. The *Smurf2* knockdown-mediated suppression of NLRP3 ubiquitination and consequent pyroptosis activation underlie the enhanced antitumor efficacy of curcumin, which is counteracted by *Smurf2* overexpression [172]. circRPPH1 is obviously overexpressed in LUAD tissues and facilitates disease progression through interaction with the transcription factor MAFK. This interaction enhances the transcription of *SIRT1*, subsequently suppressing pyroptosis. Experimental validation confirms that circRPPH1 silencing induces pyroptosis and significantly inhibits tumor growth [173]. In the realm of radiotherapy, investigations into the RSPO3-β-catenin-NF-κB-NLRP3 signaling pathway have revealed its critical role in regulating pyroptosis and modulating NSCLC radiosensitivity. As a transcriptional regulator of RSPO3, FOXP3 is a potential synergistic target to enhance radiotherapy outcomes when combined with RSPO3 modulation [174]. Nonetheless, innovative photodynamic therapy approaches warrant consideration. A research team designed a human cell membrane vesicle-based nano platform (HCNP) capable of inducing cell death of LUAD through caspase-3/GSDME-mediated pyroptosis. This platform employs VSVG protein-mediated tumor cell membrane anchoring to deliver light-activated ROS, which disrupts mitochondrial function and triggers pyroptosis [175].

### Leukaemia

Leukaemia, a clonal malignancy of hematopoietic stem cells, remains a significant clinical challenge due to treatment resistance and severe adverse effects associated with conventional therapies such as chemotherapy and allogeneic hematopoietic stem cell transplantation. Recent studies have revealed that chemoresistance in acute myeloid leukemia (AML) is mechanistically linked to dysregulated necroptosis pathways. Specifically, elevated expression of the NF-κB target gene TNFAIP3/A20 has been shown to suppress RIPK1-dependent necroptosis in AML cells, positioning A20 as a potential therapeutic target [176]. This finding is further supported by evidence showing that R-2-hydroxyglutarate (R-2HG) in IDH-mutant AML cells induces necroptosis through RIPK1 upregulation. Cooperated with demethylating agents, which effectively restore RIPK3 expression, offering a novel therapeutic option [177]. In the field of nanomedicine, the smart nanoparticle Cas-CMV@LM achieves precise bone marrow delivery via CXCR4-overexpressing mesenchymal stem cell-derived vesicles, triggering leukemic blast necroptosis and markedly suppressing disease progression in patient-derived xenograft models [178].

The pyroptosis signaling pathway has also emerged as a critically novel therapeutic possibility in leukemia management, and its molecular components and inflammatory mediators exhibit strong correlations with disease progression and prognosis. For instance, S100A8/A9 heterodimers have been shown to drive GSDMD-dependent pyroptosis in acute lymphoblastic leukemia (ALL), while FLOT1 promotes AML progression through dual suppression of pyroptosis and apoptosis [179, 180]. Notably, the erlotinib-gold(I) complex EG2 induces AML cell differentiation into DCs and triggers non-canonical pyroptosis—collectively reducing MDSCs, expanding CD4^+^/CD8^+^ T cells and CD69-activated T cells, and decreasing regulatory T cell populations to reshape the immunosuppressive milieu [181]. Combination therapy with PDE3A inhibitor anagrelide and idarubicin has shown synergistic anti-leukemic effects through caspase-3-GSDME-mediated pyroptosis in PDE3A-high AML [182]. In vivo validation of the HDAC inhibitor chidamide revealed its therapeutic potential in T-LBL/ALL through activation of the FOXO1-caspase 3-GSDME pathway [163]. Tyrosine kinase inhibitors (TKIs) are therapeutic agents for chronic myeloid leukemia (CML). To address the resistance to TKIs, researchers designed 5-FU@HFn nanoparticles loaded with 5-FU by recombinant human heavy chain ferritin nanocages. Its combination with DAC effectively inhibited leukemia progression by activating GSDME-induced pyroptosis [183]. The antiparasitic agent chloroquine (CLQ) in combination with Bcl-2 inhibitor venetoclax (VEN) exhibits robust preclinical activity against multiple myeloma and leukemia through IFIT1/IFIT3-mediated pyroptosis [184]. Most recently, the endogenous antimicrobial peptide Hs02 has been identified as a novel therapeutic candidate for its ability to modulate pyroptosis-related components and induce HL-60 cell death, demonstrating significant anti-leukemic potential [185]. The integration of molecular pathway modulation with nanotechnology platforms represents a paradigm shift in precision oncology, offering new hope for patients with refractory leukemia subtypes.

### Melanoma

Melanoma cells show low levels of RIPK3 and CYLD, making them naturally resistant to necroptosis [117]. To overcome this, potential treatments might involve increasing RIPK3 expression or combining caspase inhibitors with radiotherapy or chemotherapy. The RNA editing enzyme ADAR1 plays a pivotal role in conferring resistance to immune checkpoint blockade (ICB) therapy by suppressing the immunogenicity of dsRNA and inhibiting the accumulation of endogenous Z-RNAs. Depletion of ADAR1 leads to the accumulation of endogenous Z-RNAs, which activate ZBP1. ZBP1 then interacts with RIPK3 and MLKL, initiating necroptosis and enhancing innate immune responses. Despite the development of ADAR1 inhibitors being at an experimental stage, small-molecule compound CBL0137 has been proven to effectively induce Z-DNA formation and activate ZBP1-dependent necroptosis in mouse melanoma models, thereby boosting adaptive immunity and improving responsiveness to immunotherapy. By circumventing ADAR1-mediated immune silencing, CBL0137 directly activates ZBP1, offering a promising strategy for overcoming cancers resistant to ICB. Additionally, the development of clinically applicable ADAR1 inhibitors represents another potential avenue [186]. Several compounds directly induce necroptosis in melanoma. The novel naphthyridine derivative 3u can induce necroptosis in various cancer cells, including melanoma A375 cells. Oregano extracts trigger necroptosis through mitochondrial and DNA damage, selectively inhibiting melanoma cell proliferation without exhibiting cytotoxicity or mutagenicity towards non-tumor proliferating cells [187, 188]. The WEE1 kinase inhibitor AZD1775 induces necroptosis, sensitizing tumor cells to anti-PD-1 treatment. Using miRNA encoding necroptosis mediator MLKL to treat melanoma cells not only induces necroptosis but also elicits robust antitumor responses, significantly enhancing tumor growth inhibition when combined with PD-1 inhibitors [189, 190].

Pyroptosis-based therapies are gaining traction in melanoma treatment. One research group developed iron-based metal-organic framework nanoparticles loaded with triptolide (TPL@TFBF), which target melanoma cells to induce ferroptosis and pyroptosis by increasing ROS levels. This process inhibits tumor growth and lung metastasis. When combined with ICB therapy, this strategy further suppresses tumor progression [191]. Additionally, certain pyroptosis inducers, such as recombinant human adenovirus 5, TREM2, and terphenyllin, have also exhibited significant antitumor effects against melanoma [192–194]. Researchers are exploring innovative treatments that use nanotechnology for co-delivering MS-275 and V-9302. Other approaches under investigation include bioengineered BRAF and COX2 inhibitor nanogels, as well as self-assembling, CXCR4-targeted pyroptosis nanotoxins [195–197]. Emerging studies suggest the pyroptosis pathway holds therapeutic promise, with hBMSC-Exos, exosomal miR-211-5p, and 12-PRlncRNA identified as central candidates [198–200].

### Gastric cancer

Gastric cancer poses an essential public health challenge due to its persistently high global incidence, ranking as the fifth most common malignancy and the third leading cause of cancer-related deaths worldwide. During intestinal-type gastric cancer progression, epithelial cells trigger necroptosis in FOLR2^+^ macrophages via APP-TNFRSF21 signaling, resulting in a substantial depletion of FOLR2^+^ macrophages. These macrophages enhance CD8^+^ T cell cytotoxicity through antigen cross-presentation, thereby contributing to anti-tumor immunity [201]. This mechanism offers key insights into how intestinal metaplasia undergoes malignant transformation. MYOSLID, a newly discovered long non-coding RNA located at chromosome 2q33.3, exhibits anti-necroptotic activity. Its elevated expression blocks programmed necrotic cell death, promoting cancer cell survival and proliferation, although the exact molecular mechanisms involved still require further investigation [202]. Derived from Jaspine B, compound C8 is a novel therapeutic agent that inhibits gastric cancer cell growth through RIPK3-dependent necroptosis. Preclinical data demonstrate that C8 induces ICD without significantly altering tumor immunity, highlighting both safety and antitumor potential [203].

The contribution of pyroptosis to gastric carcinogenesis is closely associated with tumor progression stages. Initially tumor-suppressive, it inhibits oncogenesis in early stages yet promotes tumor growth in advanced disease by sustaining the inflammatory microenvironment. GSDMB exemplifies this context-dependent functionality—although it exhibits oncogenic properties in gastric cancer, CTLs still activate it via granzyme A secretion to trigger pyroptosis in gastrointestinal epithelial tumors, thereby eliciting measurable anti-tumor effect [204]. Although the underlying mechanisms require further exploration, pyroptosis still holds significant therapeutic potential in gastric cancer due to its capacity to activate the immune system. Recent therapeutic advances include the development of IBI315, a novel recombinant human IgG1 bispecific antibody that simultaneously targets PD-1 and HER2. This innovative agent works through a complicated mechanism: one arm binds HER2 on tumor cells while the other activates PD-1 on T cells, triggering granulysin A secretion. The released granulysin A then cleaves GSDMB to induce pyroptosis. Preclinical data showing 117% and 77% tumor volume reductions in N87 and PDX-1 models, respectively, position IBI315 as a promising immunotherapy candidate for HER2-positive gastric cancer [205]. Combination therapies targeting pyroptosis exhibit synergistic effects. A notable example involves co-administering the BCL-2/BCL-XL inhibitor APG-1252 with lapatinib, a HER2-targeted agent that enhances GSDME expression. This combination demonstrated 17.74% tumor growth suppression in preclinical HER2-positive gastric cancer models [206]. Regarding conventional chemotherapeutics, 5-FU sensitivity is regulated by ALKBH4 through an epigenetic mechanism. ALKBH4 suppresses GSDME transcription, thereby inhibiting pyroptosis and diminishing chemosensitivity to 5-FU. ALKBH4 knockdown reverses this effect by restoring pyroptosis and significantly enhancing 5-FU responsiveness. This finding positions ALKBH4 as a promising therapeutic target for improving chemotherapy outcomes [207]. While *Helicobacter pylori* (HP) infection is a well-established risk factor for gastric carcinogenesis, recent studies reveal a paradoxical therapeutic benefit: HP-infected gastric cancer cells exhibit heightened sensitivity to 5-FU. Mechanistically, HP enhances chemosensitivity through pyroptosis activation mediated by the NF-κB/METTL3/BRD2/FOXO4-FLIP/Caspase-8 signaling pathway. Thus, BRD2 may become a promising therapeutic target for enhancing chemotherapy efficacy in gastric cancer [208]. On the other hand, HP infection triggers gastric atrophy (AG) through VacA-mediated TNFAIP3 activation, which stabilizes TRAF1 to drive pyroptosis. AG is a key pre-cancerous lesion in gastric cancer, and this study may be a new strategy for treating AG, which in turn may provide a new strategy for inhibiting the progression of gastric cancer [209].

### Glioma

Gliomas constitute the most common malignant central nervous system tumors. High-grade variants carry grave prognoses, with a demonstrated 5-year survival rate of just 6.9% in patients [210]. Recent drug development breakthroughs identify the sinomenine derivative 7I as a potent anti-glioblastoma agent, with demonstrated anti-glioblastoma efficacy across in vitro and in vivo preclinical models. Mechanistically, 7I elicits necroptosis via the canonical RIPK1/RIPK3/MLKL pathway while simultaneously activating the p53/p21/CDK1/cyclin B pathway to enforce G2/M arrest, thereby achieving potent tumor suppression and positioning 7I as a therapeutic candidate for glioblastoma (GBM) treatment [211]. In parallel, ultrasound-activated IL-12mRNA@cRGD-CM-CaCO_3_NPs release CO_2_, which induces necroptosis in tumor cells and promotes elevated intratumoral IL-12 expression, IFN-γ production, and CD8^+^ T-cell infiltration [212]. Glioma stem cells (GSCs), key contributors to therapeutic resistance and poor prognosis, exhibit selective vulnerability to TAK1 kinase inactivation. CRISPR-based functional genomics confirms that TAK1 depletion activates RIPK1-dependent apoptosis pathways in GSCs [213]. In addition, cysteine- and glycine-rich protein 2 (CSRP2) is consistently overexpressed in glioma tissues and cell lines, where it drives malignant progression through dual oncogenic mechanisms. CSRP2 drives tumor progression by activating the JAK-STAT1 signaling pathway, enhancing cellular proliferation, migration, and invasive capabilities. Concurrently, this protein exerts inhibitory effects on necroptosis [214].

Pyroptosis has emerged as a key therapeutic mechanism in glioma treatment, particularly when combined with conventional therapies, immunotherapies, or nano delivery platforms. The nanocomplex CuCCT@CM-Ang—composed of polyethylene glycol, cisplatin, copper ions, tannic acid, and catalase—effectively penetrates the blood-brain barrier and selectively targets tumor sites, demonstrating high tumor tissue accumulation. This system potently induces pyroptosis in GBM cells while synergistically enhancing immunotherapy efficacy [215]. RT remains a cornerstone treatment for GBM; however, GOLPH3L is markedly upregulated in radioresistant GBM, where it binds to STING, suppresses its activation, and diminishes pyroptosis. This process collectively impairs the TME and contributes to therapeutic resistance. A dual-targeting strategy combining the GOLPH3L inhibitor VB5 with the STING agonist ADU-S100 effectively restores pyroptotic cell death and counteracts radioresistance, offering a promising therapeutic avenue [216]. Similarly, tertiary amine-modified triterpenoid nanoparticles achieve mitochondrial-specific targeting, inducing pyroptosis-mediated tumor cell death [217]. Harnessing the immunostimulatory potential of bacterial components and pyroptosis, researchers engineered an immunoreactive self-lytic *Salmonella*-based nanodelivery system (IASNDS). This platform synergizes *Salmonella*-derived delivery vectors with lytic nanocapsules, exploiting dual immunostimulation from Gram-negative bacterial elements and pyroptosis. The system effectively expands tumor-infiltrating lymphocytes, resulting in a 6.62-fold increase in CD4^+^ T cells compared to controls, along with elevated populations of CD8^+^ T cells and Granzyme B^+^ cytotoxic cells, as well as enhanced pro-inflammatory cytokine expression. This dual-action mechanism effectively suppresses postoperative GBM recurrence [218]. Building on these advances, multimodal strategies such as near-infrared II photoacoustic imaging-guided with chemo-photothermal immunotherapy have emerged for pyroptosis-driven GBM therapy. This approach combines precise tumor visualization, thermal ablation, and immune activation to target orthotopic GBM through coordinated pyroptotic cell death pathways [219, 220].

## Pyroptosis and necroptosis as a promising strategy in cancer therapy

The core value of pyroptosis and necroptosis in the field of oncology therapeutics lies in the release of pro-inflammatory cytokines such as IL-1β and IL-18, along with DAMPs, which collectively contribute to DCs activation and the priming of CTLs [3]. By remodeling the immunosuppressive TME, these pathways elicit potent immune-stimulatory effects and circumvent resistance commonly associated with traditional apoptotic pathways [71]. This mechanism highlights pyroptosis and necroptosis as potential therapeutic avenues through synergistic strategies: either combining with conventional chemotherapeutic agents to reverse chemoresistance, or pairing with ICIs to overcome the inefficacy of immunotherapy in immunologically inert tumors [3, 221]. Emerging preclinical and clinical studies have provided validation for this perspective. For instance, the RIPK1 inhibitor GSK0547 significantly suppresses tumor growth in vivo, promotes M1 macrophage polarization, and reprograms TAMs to activate CD4^+^ Th1/Th17 and CD8^+^ T cells, and demonstrates synergistic efficacy in combination with PD-1 inhibitors or ICOS agonists, resulting in significantly prolonged survival [101]. Docosahexaenoic Acid (DHA) activates caspase-1–mediated pyroptosis, and a clinical trial (NCT03831178) confirmed its utility in the neoadjuvant treatment of female breast cancer patients, mitigating alterations in hematologic parameters, preventing immune cell exhaustion, and augmenting ex vivo cytokine secretion upon stimulation, ultimately leading to an improvement in systemic immune function [222, 223]. Similarly, FL118 exerts its tumor-suppressive effects by activating the NLRP3 inflammasome, which promotes caspase-1 activation and upregulates GSDMD expression, thereby inhibiting the proliferation, migration, and metastasis of CRC and pancreatic cancer cells [223, 224]. A related clinical trial (NCT06206876) is currently underway to further evaluate its therapeutic potential in patients with advanced PDAC [225]. The combination of Decitabine and Paclitaxel reverses methylation of the GSDME enhancer, thereby increasing GSDME expression and restoring chemosensitivity to paclitaxel in MCF-7/paclitaxel-resistant BC cells, currently under investigation in a clinical trial (NCT03282825) [226]. Nanocarrier technology is a promising direction that offers potential advantages such as improved drug bioavailability and reduced side effects through targeted delivery. For example, a ZIF-8 nanoparticle system surface-functionalized with anionic hyaluronic acid (HA) not only facilitates the shift from apoptosis to pyroptosis but also disrupts immune evasion mediated by MDSCs [227]. Collectively, inducing pyroptosis or necroptosis represents a promising immunostimulatory strategy to counteract resistance to conventional cancer therapies.

## Conclusions

Pyroptosis and necroptosis, as major forms of ICD, exhibit a dualistic “double-edged sword” role in cancer immunotherapy. This review systematically summarizes their molecular mechanisms, regulatory effects on the TME, and therapeutic potential across multiple cancer types, elucidating potential mechanisms of cancer immunotherapy resistance. However, accumulating evidences indicate that, especially during chronic inflammation, these processes can promote tumor progression. Although preclinical studies demonstrate that combining ICIs with pyroptosis-inducing agents can effectively convert immunologically “cold” tumors into “hot” ones, significant challenges persist in translating these results to clinical practice. Firstly, tumor heterogeneity poses a major challenge, as the susceptibility to pyroptosis or necroptosis varies across cancer types and subtypes, requiring personalized strategies based on tumor-specific molecular and microenvironmental features. Secondly, excessive inflammation raises safety concerns, potentially causing tissue damage or fostering tumor-promoting chronic inflammation; for example, MLKL overexpression during necroptosis can upregulate CD47 via IL-6 signaling to inhibit macrophage phagocytosis, while lactate from pyroptosis may drive M2 macrophage polarization. Therefore, the development of spatiotemporally controlled delivery systems, such as pH- or ROS-responsive nanoparticles, is essential to mitigate off-target toxicity while maintaining therapeutic efficacy. A further challenge is the development of drug resistance, as tumor cells may escape PCD via epigenetic silencing—such as *RIPK3* promoter methylation—or metabolic reprogramming, including glycolysis-driven inhibition of necroptotic signaling. Targeting compensatory resistance pathways—such as the SAMHD1-STING axis—or combining necroptosis/pyroptosis inducers with epigenetic modulators like decitabine could represent promising strategies to overcome these barriers.

Looking forward, therapeutic strategies leveraging pyroptosis and necroptosis should prioritize combination treatments and precise modulation of cell death pathways, with nanotechnology providing promising avenues for targeted drug delivery. By enabling controlled activation of Gasdermin or RIPK1/3–MLKL pathways, such platforms can enhance tumor-specific immune activation while limiting systemic diffusion of DAMPs and minimizing collateral damage to normal tissues. By virtue of their modular design, nanocarriers enable the co-delivery of chemotherapeutic agents, immune agonists, and epigenetic modifiers, orchestrating a synergistic “death-immunity-epigenetic” axis to combat drug resistance and enhance therapeutic efficacy. In conclusion, pyroptosis and necroptosis have introduced new dimensions to tumor immunotherapy. However, their dual functionality demands a balanced approach that integrates mechanistic insights, technological innovation, and clinical translation. Ongoing advancements in this field offer promising avenues to overcome cancer immunotherapy resistance, discover new therapeutic targets, and develop next-generation precision oncology strategies.

## Data Availability

No datasets were generated or analysed during the current study.

## Abbreviations

ICD: Immunogenic cell death'
YARS: Tyrosine aminoacyl-tRNA synthetase
TME: Tumor microenvironment
ER: Estrogen receptor
MDSC: Myeloid-derived suppressor cell
PR: Progesterone receptor
ICIs: Immune checkpoint inhibitors
HER2: Epidermal growth factor receptor 2
DCs: Dendritic cells
MIT: Mitoxantrone
PCD: Programmed cell death
GA: Garcinol
DAMPs: Damage-associated molecular patterns
UCP1: Uncoupling Protein 1
CTLs: Cytotoxic T Cells
HCC: Hepatocellular carcinoma
RIPK1: Receptor-interacting serine/threonine-protein kinase 1
ICC: Intrahepatic cholangiocarcinoma
RIPK3: Receptor-interacting serine/threonine-protein Kinase 3
NEK7: NIMA-related kinase 7
MLKL: Mixed-lineage kinase domain-like protein
DLBCL: Diffuse large B-Cell lymphoma
TNF-α: Tumor necrosis factor-α
PTCL: Peripheral T-Cell lymphoma
RHIM: RIP homotypic interaction motif
HDAC: Histone deacetylase
ZBP1: Z-DNA binding protein 1
MCL: Mantle cell lymphoma
mtDNA: Mitochondrial DNA
T-LBL: T-Cell lymphoblastic lymphoma
CRC: Colorectal cancer
NSCLC: Non-Small cell lung cancer
TAMs: Tumor-Associated macrophages
LUAD: Lung adenocarcinoma
PRRs: Pattern recognition receptors
PKP3: Plakophilin 3
ASCs: Apoptosis-associated speck-like proteins
HCNP: Human cell membrane vesicle-based nano platform
CARD: Caspase activation and recruitment domains
AML: Acute myeloid leukemia
PYD: Pyrin domains
R-2HG: R-2-Hydroxyglutarate
PAMPs: Pathogen-associated molecular patterns
ALL: Acute lymphoblastic leukemia
FXR: Farnesoid X receptor
TKIs: Tyrosine kinase inhibitors
EMT: Epithelial-mesenchymal transition
CML: Chronic myeloid leukemia
HHT: Homoharringtonine
CLQ: Chloroquine
DSS: Dextran sulfate sodium
VEN: Venetoclax
CaZCH NPs: Calcium-based nanoinducers
ICB: Immune checkpoint blockade
PDAC: Pancreatic ductal adenocarcinoma
HP: Helicobacter pylori
MET: Macrophage extracellular trap
GBM: Glioblastoma
PGE2: Prostaglandin E2
GSCs: Glioma stem cells
BC: Breast cancer
CSRP2: Cysteine- and glycine-rich protein 2
TNBC: Triple-negative breast cancer
DHA: Docosahexaenoic acid
AQP1: Aquaporin 1
HA: Hyaluronic acid
BAD: Bcl-2-associated death promoter
